# Fresh Perspectives

**Published:** 2008-09-15

**Authors:** Lynne S. Wilcox

This issue of *Preventing Chronic Disease* (*PCD*) offers fresh perspectives on the relationships among life stages, health behavior, and chronic disease. We include several reports from a survey of childbearing women in Cameron County, Texas, and Matamoros, Tamaulipas, Mexico, designed to support action by the United States-Mexico Border Health Association (1). *PCD* has addressed border health previously and observed that the communities of the US-Mexico border region share a culture with health concerns that are different from other places in either country (2).

In this issue, chronic disease experts will note the connections between reproductive health and overall women's health. When the United States-Mexico Border Health Commission established 2010 objectives for reproductive health and chronic disease, it recognized that baseline data were not available. The Brownsville-Matamoros Sister City Project for Women's Health (BMSCP) examined the feasibility of meeting this need in reproductive health (1).

In the United States, 14.2 million women are aged 18 to 24 years (3). According to data from the 2005 Behavioral Risk Factor Surveillance Survey, 23% of women in this age group reported not exercising during the previous 30 days, 24% were current smokers, 38% had a body mass index (BMI) of 25 or greater, and 34% reported binge drinking (consuming more than 5 drinks at one time) during the past month (3). Of women in the same age group, 18% had been told they have asthma, 1% diabetes, and 4% high blood pressure. Nine percent reported their health as only fair to poor (3).

Mexico's National Survey of Health and Nutrition estimated that 19.7 million women aged 10 to 29 years were living in Mexico in 2006 (4). Ten percent of Mexican women aged 16 to 19 years and 11% of women aged 20 to 29 years had smoked 100 cigarettes or more in their lifetimes. Thirty-three percent of young women aged 12 to 19 years and 54% of women aged 20 to 29 years had a BMI of 25 or more. Among women aged 16 to 19 years, 14% reported ever drinking and 31% of these women reported binge drinking at least once per month (4). Other reports have found that 18% of Mexican women aged 18 to 29 years are inactive (5). Among Mexican women aged 20 to 29 years, 3% have diabetes and 12% have high blood pressure (6).

We know the numbers, but how do we reach these women? The primary reason US women aged 15 to 44 years enter the hospital is to give birth (7). Women in this age group most commonly cite prenatal and gynecologic care as the reasons they visit a doctor (8). Among Mexican women aged 20 years or older, 19% of all hospitalizations are for childbirth (4). These encounters offer opportunities for preventing and treating chronic diseases.

But the health care system cannot be our only partner. In the United States, the lack of health insurance among 31% of women and 36% of men aged 18 to 24 years restricts their opportunities to obtain clinical care (3). In fact, young adults are more likely to lack health care coverage than any other age group. Continuing to work in the broader community to encourage good health over the life span is essential.

I close this editorial with acknowledgement of my own opportunity for fresh perspectives. After serving for 6 years as the founding editor of *PCD*, I am retiring from the United States Public Health Service to pursue new adventures. It has been a privilege and a delight to work with the *PCD* production team, our editorial board, and our guest editors, authors, and reviewers. From all of you I have learned the power of the pen. I encountered new ideas with every issue and leave with an enhanced understanding of the vast scope of our work.

I am grateful for all the support you have given *PCD* and its staff as we establish a new channel for scientific communication in preventing chronic disease. I cherish my relationships with the committed public health workers I have met through this journal. From my perspective, you are the quiet heroes.
